# Oral VV261 administration protects mice from lethal Crimean-Congo hemorrhagic fever virus challenge

**DOI:** 10.1128/jvi.01583-25

**Published:** 2025-11-25

**Authors:** Xi Wang, Huan Xu, Liushuai Li, Fan Wu, Jiang Li, Jingshan Shen, Gengfu Xiao, Wei Zheng, Leike Zhang, Zhihong Hu, Manli Wang

**Affiliations:** 1 State Key Laboratory of Virology and Biosafety, Wuhan Institute of Virology, Chinese Academy of Sciences 74614, Wuhan, China; 2 Department of Pediatrics, Union Hospital, Tongji Medical college, Huazhong University of Science and Technology 12443, Wuhan, China; 3 State Key Laboratory of Drug Research, Shanghai Institute of Materia Medica, Chinese Academy of Sciences 58298, Shanghai, China; 4 Vigonvita Shanghai Co., Ltd, Shanghai, China; St Jude Children's Research Hospital, Memphis, Tennessee, USA

**Keywords:** Crimean-Congo hemorrhagic fever virus, nucleoside analog, VV261, oral administration

## LETTER

Crimean-Congo hemorrhagic fever virus (CCHFV), a member of the family *Nairoviridae* within the class *Bunyaviricetes* (1), is distributed across more than 30 countries and poses a significant threat to global health. CCHFV infection causes acute viral hemorrhagic fever with a high case fatality rate (10–40%) (2, 3). No FDA-approved therapeutics exist, prompting the WHO to list it as a priority pathogen for research and development since 2017. While some antiviral compounds like T-705 (favipiravir) (4), H44 (5), and baloxavir sodium (6) have shown promise in animal models, drug development for CCHFV needs acceleration.

VV261, an oral double prodrug of 4′-fluorouridine (4′-FU), exhibits improved stability and pharmacokinetics. It has demonstrated efficacy against related bunyaviruses such as Severe fever with thrombocytopenia syndrome virus (7). VV261 has entered phase I clinical trials in China. Given that 4′-FU targets the RNA-dependent RNA polymerase (RdRp) and shows broad-spectrum anti-RNA virus activity (8–13), we evaluated the antiviral potential of VV261 against CCHFV.

**Fig 1:**
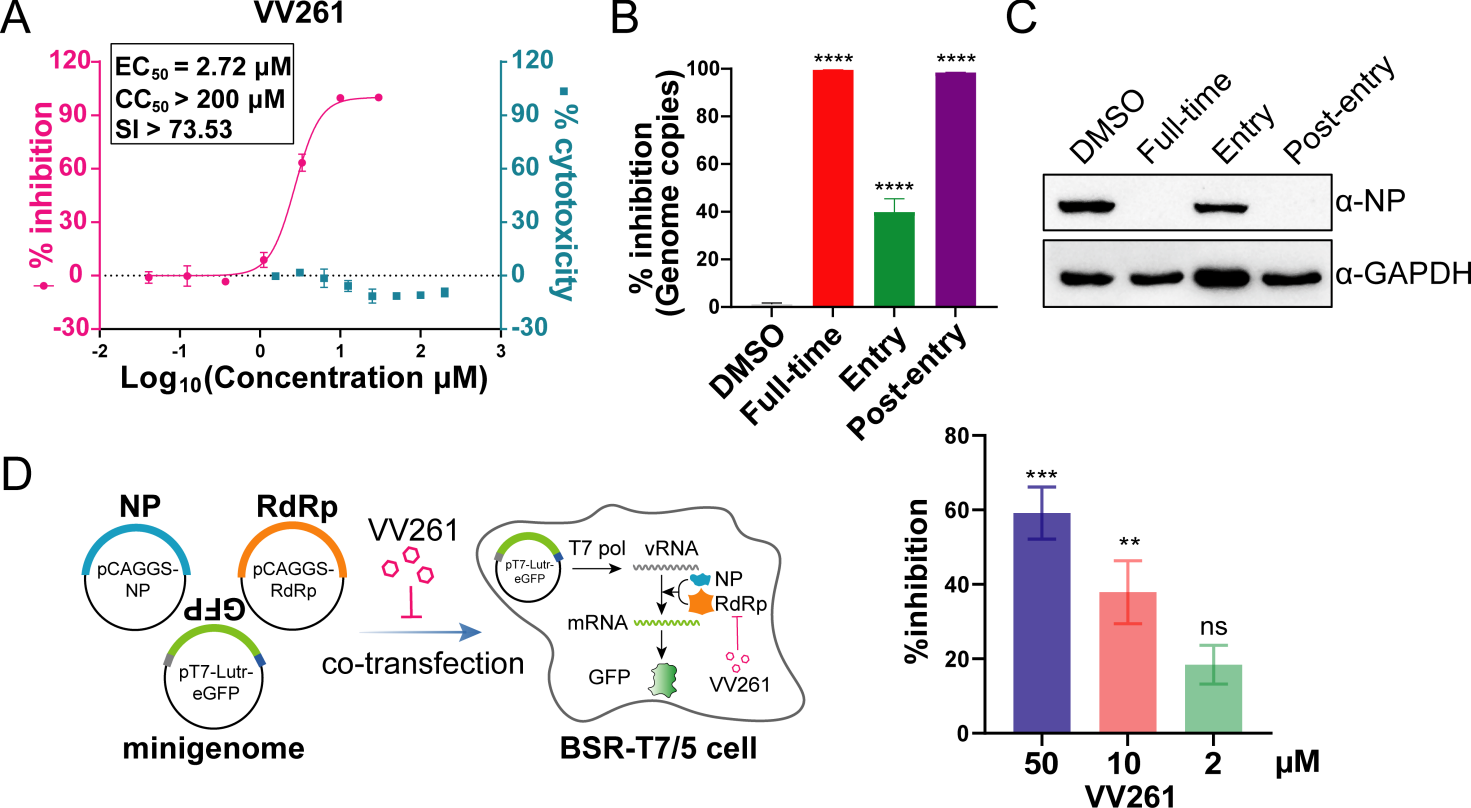
The nucleoside analog VV261 is potent in inhibiting CCHFV infection *in vitro*. (**A**) EC_50_ assay of VV261 against CCHFV. Serially diluted VV261 was added to HUVECs and the cells were infected with CCHFV at an MOI of 0.1. The progeny virus yield was quantified using qRT-PCR, and the EC_50_ values were calculated. The cytotoxicity of VV261 on HUVECs was determined by the CCK-8 assay. Data represent three biological replicates, and error bars represent the standard deviation of the mean. (**B and C**) VV261 inhibits virus infection at post-entry stage. HUVEC cells were infected with CCHFV at an MOI of 0.1, and VV261 (20 µM) was added to cell culture during “Full-time,” “Entry,” or “Post-entry” stages. The viral genome copies from the culture supernatant were quantified by qRT-PCR, and the inhibition ratio was calculated (**B**), viral NP expression level from each treatment was determined by western blot (**C**). (**D**) VV261 inhibits CCHFV RdRp activity in a mini-replicon system. Co-transfection of a T7 promoter-driven GFP minigenome plasmid with two helper plasmids expressing CCHFV NP and RdRp into BSR-T7/5 cells (which stably express T7 RNA polymerase) was performed. Driven by RdRp and NP, the minigenome transcribes GFP mRNA, resulting in GFP expression within the cells. VV261 inhibited GFP expression by targeting to RdRp in a dose-dependent manner. Statistical significance was analyzed by one-way ANOVA with Dunnett’s multiple-comparison test for (**B**) and (**D**). Statistical significance was assigned when **P < 0.01, ***P < 0.001, ****P < 0.0001, ns, not significant.

We first tested the anti-CCHFV activity of VV261 *in vitro*. In human umbilical vein endothelial cells (HUVECs), VV261 potently inhibited CCHFV infection with a median effective concentration (EC_50_) value of 2.72 ± 0.28 µM (Fig. 1A). It showed negligible cytotoxicity as the 50% cytotoxicity concentration (CC_50_) value is greater than 200 µM, resulting in a high selective index (SI > 73.53) (Fig. 1A). These data demonstrated that VV261 is a potent inhibitor against CCHFV *in vitro*.

We next performed a time-of-addition assay to investigate which stage of the viral life cycle VV261 targets to inhibit CCHFV infection. Treatment during the post-entry phase or for the full duration nearly completely suppressed progeny virus production (Fig. 1B) and viral nucleoprotein (NP) expression (Fig. 1C). Some inhibition during the entry stage was likely attributed to the residual compound retained within the cells. Taken together, these results indicate that VV261 mainly targets the post-entry stage of CCHFV infection.

To further investigate the inhibitory activity of VV261 against viral transcription and replication machinery—specifically, the RdRp, a previously established mini-replicon system was employed (6). We found that VV261 dose-dependently reduced GFP expression by approximately 20%, 38%, and 59% at concentrations of 2, 10, and 50 µM, respectively (Fig. 1D), suggesting the inhibition of RdRp activity, consistent with its mechanism as a nucleoside analog.

**Fig 2:**
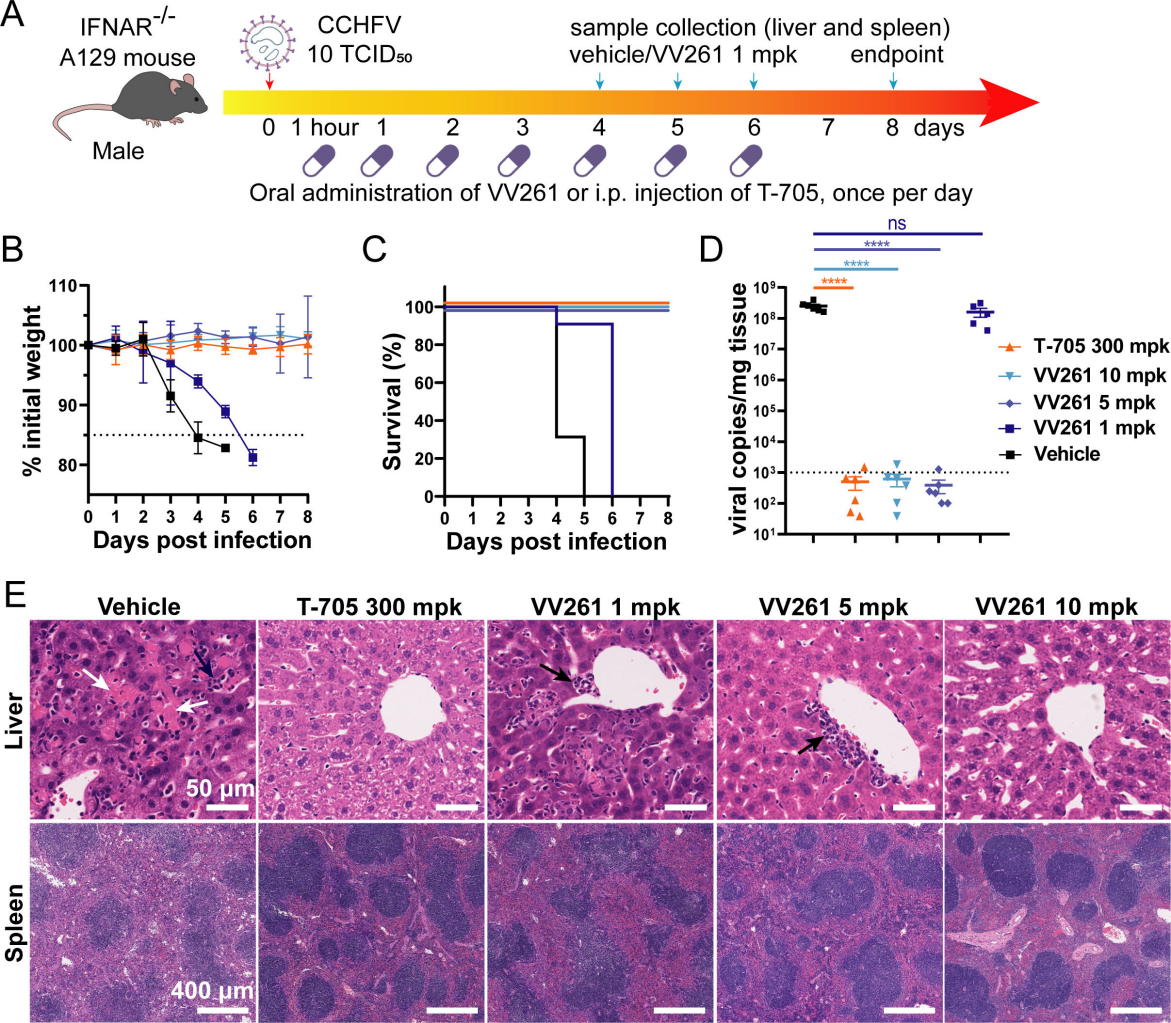
Oral administration of VV261 inhibits CCHFV infection and alleviates pathology *in vivo*. (**A**) A graphical illustration of animal experiment design. Ten-week-old male A129 mice (type I interferon receptor knockout, *n* = 6 mice/group). Each mouse was infected with CCHFV at a dose of 10 TCID_50_ via the i.p. route. One hour post-infection, mice were administered with T-705 (positive control) at 300 mg/kg via the i.p. route or orally administered with VV261 at 1, 5, and 10 mg/kg once daily, while the control group received vehicle solution orally. The mice were monitored daily and euthanized when they exhibited a weight loss of over 15% of the starting weight or at the end of the experiment. Weight loss curves (**B**) and survival curves (**C**) were plotted according to the data obtained daily. Dashed line in (**B**) indicates 85% of initial weight. (**D**) Viral loads in liver tissue were determined by qRT-PCR. Dashed line indicates limit of detection. Statistical significance was analyzed by one-way ANOVA with Dunnett’s multiple-comparison test, and significance was assigned when ****P < 0.0001, ns, not significant. (**E**) Histopathological analysis of mice liver and spleen. White arrows and black arrows indicated the necrosis of hepatocytes and lymphocyte filtration in the liver, respectively. Scale bar, 50 µm in liver, 400 µm in spleen.

The anti-CCHFV efficiency of VV261 was further evaluated *in vivo* using A129 mice, which are deficient in IFNα/β receptor and are highly susceptible to lethal CCHFV challenge (14). Mice were challenged intraperitoneally (i.p.) with 10 TCID_50_ CCHFV. One hour post-infection, different doses of VV261 (1, 5, and 10 mg/kg/day [mpk]) were administered orally (Fig. 2A). Mice in the negative control group received the drug vehicle orally, while the positive control group was treated with T-705 (300 mpk) via i.p. injection. Following the initial administration, the treatment continued for 6 days.

Vehicle-treated mice lost weight at 2 days post-infection (Fig. 2B) and ultimately succumbed to the virus infection within 5 days (Fig. 2C). In contrast, mice receiving 10 or 5 mpk VV261 or T-705 showed no significant weight loss or clinical signs (Fig. 2B), and all survived (Fig. 2C). The low-dose VV261 (1 mpk) delayed weight loss and extended survival (Fig. 2B and C), although it did not provide full protection. Viral loads in the livers of mice treated with T-705, VV261 (5 mpk), and VV261 (10 mpk) were nearly undetectable (Fig. 2D), suggesting potent suppression of viral replication *in vivo*. We also got similar viral load results in spleen tissue (Fig. S1). In contrast, the 1 mpk dose of VV261 was ineffective (Fig. 2D; Fig. S1), which aligns with the pharmacokinetic data (7) showing its maximum concentration (*C*_max_ ~ 2.70 µM) barely reaches its EC_50_ value (2.72 µM).

Pathology examination of major target organs (15) showed that T-705, 5 mpk, and 10 mpk VV261 treatments remarkably alleviated tissue damage, reducing hepatocellular necrosis (white arrows) and lymphocyte filtration (black arrows) in the liver, as well as less disruption of splenic structure in spleen (Fig. 2E).

In summary, we have demonstrated that VV261 inhibits CCHFV replication efficiently *in vitro* and *in vivo*. Its mechanism involves targeting RdRp transcription activity. A dosage of 5–10 mpk of VV261 conferred 100% protection in a lethal mouse model, comparable to a higher dose of T-705 (300 mpk). However, this study has several limitations worthy of further research. For instance, evaluating the drug’s efficacy when administering at different infection stages, extending the observation period to monitor potential disease rebound, using immunocompetent animal models will better inform the clinical translation of VV261. In addition, whether CCHFV would develop resistance to VV261 as reported in other viruses (16, 17) remains under investigation. Nevertheless, with its oral bioavailability and favorable dosing regimen, VV261 represents a promising candidate for treating CCHFV infection.

## SUPPLEMENTAL MATERIAL

Supplemental material jvi.01583-25-s0001.docx Supplemental methods and Fig. S1.

ASM does not own the copyrights to Supplemental Material that may be linked to, or accessed through, an article. The authors have granted ASM a non-exclusive, world-wide license to publish the Supplemental Material files. Please contact the corresponding author directly for reuse.
