# Supplementary material for: Oral VV261 administration protects mice from lethal Crimean-Congo hemorrhagic fever virus challenge
Source: J Virol. 2025 Nov 25;99(12):e01583-25. doi: 10.1128/jvi.01583-25 (PMC12724320; doi:10.1128/jvi.01583-25)
Supplement: Supplemental material — Supplemental methods and Fig. S1. [file jvi.01583-25-s0001.docx]

***Journal of Virology***

**Supplementary Materials**

**Oral VV261 administration protects mice from lethal Crimean-Congo hemorrhagic fever virus challenge**

Xi Wang ^a#^, Huan Xu ^a,b#^, Liushuai Li ^a^, Fan Wu ^a^, Jiang Li ^a^, Jinshan Shen ^d^, Gengfu Xiao ^a^, Wei Zheng ^c*^, Leike Zhang ^a*^, Zhihong Hu ^a*^, Manli Wang ^a*^

^a^ State Key Laboratory of Virology and Biosafety, Wuhan Institute of Virology, Chinese Academy of Sciences, Wuhan, 430071, China

^b^ Department of Pediatrics, Union Hospital, Tongji Medical college, Huazhong University of Science and Technology, Wuhan, 430022, China

^c^ Vigonvita Shanghai Co., Ltd, Shanghai 201210, China.

^d^ State Key Laboratory of Drug Research, Shanghai Institute of Materia Medica, Chinese Academy of Sciences, Shanghai, 201203, China.

^#^**These authors contributed equally:**

Xi Wang, Huan Xu

***Corresponding author:**

Wei Zheng, Dr. Mailing address: Vigonvita Shanghai Co., Ltd., Shanghai, 201210, P. R. China. Email: wei.zheng@vigonvita.cn. Tel/Fax: +86-021-50200777. ORCID: 0009-0003-2398-7610

Leike Zhang, Dr. Prof. Mailing address: Wuhan Institute of Virology, Chinese Academy of Sciences, Wuhan 430071, P.R. China. Email: zhangleike@wh.iov.cn. Tel/Fax: +86-27-87998735. ORCID: 0000-0002-2593-2571

Zhihong Hu, Dr. Prof. Mailing address: Wuhan Institute of Virology, Chinese Academy of Sciences, Wuhan 430071, P.R. China. Email: huzh@wh.iov.cn. Tel/Fax: +86-27-87998916. ORCID: 0000-0002-1560-0928

Manli Wang, Dr. Prof. Mailing address: Wuhan Institute of Virology, Chinese Academy of Sciences, Wuhan 430071, P.R. China. Email: wangml@wh.iov.cn. Tel/Fax: +86-27-87998086. ORCID: 0000-0001-8701-3530

**Materials and methods**

**Cells and Virus**

Human umbilical vein endothelial cells (HUVEC) and BSR-T7/5 cells (Baby hamster kidney cells stably expressing T7 RNA polymerase) were grown in Dulbecco’s modified Eagle’s medium (DMEM) supplemented with 10% fetal bovine serum (FBS) in a 37 ^o^C cell incubator with 5% CO_2_. The CCHFV-IbAr10200-GFP strain (1) was deposited from the National Virus Resource Center of China (ID: IVCAS 6.9295).

**Chemical compounds**

VV261 was synthesized as previously reported (2). VV261 was dissolved in dimethyl sulfoxide (DMSO) and vehicle solution for *in vitro* and *in vivo* evaluation, respectively. One Milliliter vehicle solution was composed of 50 µl DMSO, 150 µl Solutol HS15, and 800 µl [20% (m/v) hydroxypropyl-β-cyclodextrin in water]. T-705 (99.97%; Cat No. S7975) was purchased from Selleck Chemicals (Houston, TX, USA) and was dissolved in saline solution.

**EC_50_ and CC_50_ assay**

To evaluate the activity of VV261, HUVEC cells (2 × 10^4^ cells/well) were pre-seeded in 48-well plates. Three-fold serially diluted VV261 (30 μM, 10 μM, 3.33 μM, 1.11 μM, 0.37 μM, 0.12 μM, and 0.04 μM) was added and incubated with HUVEC cells for 1 h at 37 ^o^C before virus infection. The cells were infected with CCHFV-IbAr10200-GFP at a multiplicity of infection (MOI) of 0.1 at 37 ^o^C for 1 h. Subsequently, the cell supernatant was discarded, and the cells were further incubated with fresh drug-containing medium. At 48 h post infection (p.i.), 100 μL supernatant was collected for quantification of progeny virus yield by quantitative real-time RT-PCR (qRT-PCR), and cells were fixed with 4% paraformaldehyde and stained with Hoechst33258 (Beyotime, Shanghai, China) to visualize nucleus. A dose-response curve was plotted from viral

RNA copies *versus* VV261 concentrations and the EC_50_ value was calculated. To measure the cytotoxicity of VV261, cell counting kit-8 (CCK-8) (Beyotime, China) was used according to the manufacturer’s instructions. Briefly, two-fold serially diluted VV261 (starting concentration as 200 μM) was added into cell culture and incubated with cells for 48 h, DMSO treatment was used as a negative control, and culture medium without cells and compounds was used as blank control. Ten µl CCK-8 reagent was added and mixed thoroughly. After 2 h incubation, the OD_450_ value was recorded with a Synergy H1 microplate reader (Bio Tek, VT, USA), and the % cytotoxicity was calculated as [(OD_450_ value of DMSO – OD_450_ value of VV261)/ (OD_450_ value of DMSO – OD_450_ value of blank control)] × 100%. The CC_50_ value of VV261 was calculated using GraphPad Software.

**Time-of-addition assay**

To explore the targeting stage of VV261 in CCHFV life cycle, a time-of-addition assay was performed as previously described with modification (3). Briefly, HUVEC cells (2 × 10^4^ cells/well in a 48-well plate) were treated with VV261 (20 μM) or DMSO at different stages of virus infection. For the "Full-time" treatment, cells were pre-incubated with VV261 for 1 hour before virus infection. This was followed by a 1-hour incubation with the virus in the presence of VV261. After this, the virus-drug mixture was removed. The cells were then washed with PBS and further cultured in a medium containing VV261 until the end of the experiment. For the "Entry" treatment, VV261 was added to the cells for 1 hour before virus infection and was maintained during the 1-hour viral attachment process. The virus-drug mixture was then replaced with fresh culture medium without VV261 until the end of the experiment. For the "Post-entry" experiment, virus was added to the cells to allow infection for 1 hour. The virus-containing supernatant was then replaced with a medium containing VV261 until the end of the experiment. The experimental conditions for the DMSO-treatment group were the same as those for the "Full-time" group. All experimental groups were infected with CCHFV-IbAr10200-GFP at a MOI of 0.1. At 48 h p.i., cell supernatant and cell lysates were collected for qRT-PCR and western blot analysis, respectively.

**Quantitative real-time PCR**

One hundred microliter cell culture supernatant was harvested for viral RNA extraction using the MiniBEST Viral RNA/DNA Extraction Kit (Takara Bio, Shiga, Japan) according to the manufacturer’s instructions. Murine livers were collected, homogenized and RNA were extracted using the RNAIso Plus kit (Takara Bio, Shiga, Japan). Then, cDNA was obtained using HiScript III RT SuperMix for qPCR (Vazyme, Nanjing, China). CCHFV S segment was quantified by qPCR with a Taq Pro Universal SYBR qPCR Master Mix kit (Vazyme, Nanjing, China) using primers: (qF)5’-TGAAGTGGAGAAAAGACATAGG-3’ and (qR) 5’-TCTACATGCTCATGGCTCACTGGG-3’.

**Western blot**

For western blot analysis, protein samples were separated on 10% SDS-PAGE and then transferred onto polyvinylidene difluoride (PVDF) membranes (Millipore). After being blocked with 5% milk in TBS buffer containing 0.05% Tween 20, the blot was probed with the mouse anti-NP antibody (1:2000 dilution, made in house) or rabbit anti-GAPDH serum (1:2000 dilution, made in house) as primary antibody, the horseradish peroxidase (HRP)-conjugated Goat Anti-Mouse or Anti-Rabbit IgG (Protein Tech Group, Wuhan, China) was used as secondary antibody. Protein bands were detected by SuperSignal West Pico Chemiluminescent substrate (Thermo Fisher Scientific, MA, USA).

**Mini-replicon assay**

The CCHFV mini-genome replication assay was performed as previously reported (1). BSR-T7/5 cells (5 × 10^4^ cells/well in 24-well plates) were incubated with serially diluted VV261 (50 μM, 10 μM, 2μM) or DMSO. After incubation for 1 h, the cells were transfected with 0.4 μg pCAGGS-RdRp, 0.4 μg pCAGGS-NP and 0.4 μg pT7-Lutr-eGFP using Lipofectamine 3000 (Thermo Fisher Scientific, MA, USA) according to the manufacturer’s instructions. The medium was discarded, and the cells were washed with PBS 4 h post-transfection. Fresh DMEM containing each concentration of VV261 was added to the cells. After 24 h incubation at 37 ^o^C with 5% CO_2_, the cells were fixed with 4%-polyformaldehyde for 15 min and washed three times using PBS. Nuclei were stained with Hoechst 33258 for 10 min. Images were captured using a high content imaging system (Perkin Elmer, VT, USA). And the ratio of GFP-expressing cells to total cells was calculated. The inhibition ratio of the drug group was normalized to that of the DMSO group to obtain the “% inhibition”. Statistical analyses were performed using One-Way ANOVA with Dunnett's multiple-comparison test, ****P* < 0.001, ***P* < 0.01, ns: not significant.

**Animal experiments**

All animal experiments were approved by the Institutional Animal Care and Use Committee of the Wuhan Institute of Virology, Chinese Academy of Sciences (ethics number: WIVAF01202301). Animal experiment was performed to evaluate the anti-CCHFV potency of VV261. The animal experiment employed 10-week-old male A129 mice (type I interferon receptor knockout; mice were mixed thoroughly and randomly divided into experimental groups of 6 animals). Each mouse was infected with CCHFV-IbAr10200-GFP strain at a dose of 10 TCID_50_ via the i.p. route. Mice were administered with T-705 (positive control, dissolved in normal saline) at 300 mg/kg via the i.p. route or orally administered with VV261 (prepared with vehicle solution) at 1, 5 and 10 mg/kg once a day, while the control group received oral administration of vehicle solution. The mice were monitored daily for clinical symptoms and body weight, and euthanized when exhibited a weight loss of over 15% of the starting weight or at the end of the experiment. The livers of the mice were collected for viral load detection. Viral load statistical analyses were performed using One-Way ANOVA with Dunnett's multiple-comparison test. *****P* < 0.0001, ns: not significant.

**Pathological analysis of tissues**

Liver and spleen samples of infected mice were fixed in 4% (w/v) paraformaldehyde and embedded in paraffin according to standard procedures and the embedded tissues were sectioned and dried in a hybrid furnace at 60 ^o^C for 2 h, and were stained with hematoxylin and eosin (H&E staining).

**Reference**

1. Liu K, Li L, Liu Y, Wang X, Liu J, Li J, Deng F, Zhang R, Zhou Y, Hu Z, Zhong W, Wang M, Guo C. 2024. Discovery of baloxavir sodium as a novel anti-CCHFV inhibitor: Biological evaluation of in vitro and in vivo. Antiviral Res 227:105890.

2. Cheng Y, Zheng W, Dong X, Sun T, Xu M, Xiang L, Li J, Wang H, Jian X, Yu J, Li P, Hu T, Tian G, Jiang X, Zhang L, Aisa HA, Xie Y, Xiao G, Shen J. 2025. Design and Development of a Novel Oral 4'-Fluorouridine Double Prodrug VV261 against SFTSV. J Med Chem 68:9811-9826.

3. Wang M, Cao R, Zhang L, Yang X, Liu J, Xu M, Shi Z, Hu Z, Zhong W, Xiao G. 2020. Remdesivir and chloroquine effectively inhibit the recently emerged novel coronavirus (2019-nCoV) in vitro. Cell Res 30:269-271.

**Supplementary figure S1.** Viral copies in the CCHFV-infected spleen. Spleen viral load was assessed by qRT-PCR. Dashed line indicates limit of detection. Statistical significance was analyzed by One-Way ANOVA with Dunnett's multiple-comparison, and significance was assigned when *****P* < 0.0001; ns, not significant.
